# Methanolic extract of *Tamarix Gallica* attenuates hyperhomocysteinemia induced AD-like pathology and cognitive impairments in rats

**DOI:** 10.18632/aging.101627

**Published:** 2018-11-12

**Authors:** Maibouge Tanko Mahamane Salissou, Yacoubou Abdoul Razak Mahaman, Feiqi Zhu, Fang Huang, Yuman Wang, Zhendong Xu, Dan Ke, Qun Wang, Rong Liu, Jian-Zhi Wang, Bin Zhang, Xiaochuan Wang

**Affiliations:** 1 Department of Pathophysiology, School of Basic Medicine, Key Laboratory of Education Ministry of China for Neurological Disorders, Tongji Medical College, Huazhong University of Science and Technology, Wuhan 430030, China; 2 Cognitive Impairment Ward of Neurology Department, The Third Affiliated Hospital of Shenzhen University, Shenzhen 518001, Guangdong Province, China; 3 Co-innovation Center of Neuroregeneration, Nantong University, Nantong JS226001, China; 4 Department of Genetics and Genomic Sciences, Icahn Institute of Genomics and Multiscale Biology, Icahn School of Medicine at Mount Sinai, New York, NY 10029, USA; * Equal contribution

**Keywords:** Alzheimer disease, *Tamarix Gallica (TG)*, homocysteine, tau, Amyloid-β (Aβ), cognitive impairments

## Abstract

Although few drugs are available today for the management of Alzheimer’s disease (AD) and many plants and their extracts are extensively employed in animals’ studies and AD patients, yet no drug or plant extract is able to reverse AD symptoms adequately. In the present study, *Tamarix gallica* (TG), a naturally occurring plant known for its strong antioxidative, anti-inflammatory and anti-amyloidogenic properties, was evaluated on homocysteine (Hcy) induced AD-like pathology and cognitive impairments in rats. We found that TG attenuated Hcy-induced oxidative stress and memory deficits. TG also improved neurodegeneration and neuroinflammation by upregulating synaptic proteins such as PSD95 and synapsin 1 and downregulating inflammatory markers including CD68 and GFAP with concomitant decrease in proinflammatory mediators interlukin-1β (IL1β) and tumor necrosis factor α (TNFα). TG attenuated tau hyperphosphorylation at multiple AD-related sites through decreasing some kinases and increasing phosphatase activities. Moreover, TG rescued amyloid-β (Aβ) pathology through downregulating BACE1. Our data for the first time provide evidence that TG attenuates Hcy-induced AD-like pathological changes and cognitive impairments, making TG a promising candidate for the treatment of AD-associated pathological changes.

## Introduction

Alzheimer’s disease (AD) is a progressive neurodegenerative disorder that represents the most common form of dementia and accounts for about 60% of all cases [1]. Approximately 47 million people were diagnosed with AD worldwide, a number that is expected to increase to 62% by 2030 [2]. Pathologically, AD is characterized by senile plaques due to abnormal accumulation of extracellular amyloid-β (Aβ) and the intracellular neurofibrillary tangles (NFTs) which are responsible for the AD neuronal loss. AD affected brains display several cellular dysfunctions reflecting enhanced oxidative stress, inflammatory process and calcium homeostasis disturbances. Most of these alterations are directly or indirectly linked to Aβ peptides. Aβ production, its molecular nature and biophysical properties condition the degenerative process, oxidative damage, synaptic dysfunction and inflammation [3]. The NFTs which are mainly made up of hyperphosphorylated tau, are a reflection of the imbalance in the kinase/phosphatase system, mainly involving glycogen synthase kinase 3β (GSK3β) and protein phosphatase 2A (PP2A) [4,5]. Many lines of evidence suggest that brain tissues from AD patients were exposed to oxidative stress such as glycoxidation, protein, lipid and DNA oxidation during the development of the disease, and this is at least via inhibiting PP2A activity and activating GSK3β [19–21].

Amyloid-β and hyperphosphorylated tau (p-tau) are the core elements of the plaques and tangles, respectively. Epidemiological and clinical studies have revealed that elevated blood homocysteine (Hcy) level is a modifiable risk factor for developing AD [6] and has been associated with both Aβ and p-tau. Hcy is a sulfur-containing amino acid and an intermediate product of the methionine cycle. Its normal level in the body are kept by its re-methylation to methionine in a reaction that requires folate, vitamin B6 and B12 as cofactors [7]. Hyperhomocysteinemia (HHcy) is conventionally defined as the rise of the blood Hcy level above 15 µmol/L [8]. This condition could result from dietary intake of excessive methionine, significant deficit in folate and B vitamins, or genetic alterations in certain enzymes of the methionine cycle [9,10]. The deleterious effects of Hcy in the brain are mediated via oxidative stress, DNA damage and activation of N-methyl-D-aspartate (NMDA) receptors [11–14]. Another potential link between high Hcy and AD is an alteration of the amyloid precursor protein (APP) metabolic pathways. Previous studies have shown that both genetic and diet-induced chronic high Hcy resulted in a significant increase in brain Aβ levels and deposition in transgenic mouse models of AD-like amyloidosis [14–17]. Also, elevated Hcy causes tau hyperphosphorylation, NFT formation, neurodegeneration and cognitive deficits [18]. Therefore, decreasing the elevated plasma Hcy could be a good intervention strategy for AD treatment. There are substantial evidences suggesting that brain tissues from AD patients are exposed to oxidative stress such as glycoxidation, protein, lipid and DNA oxidation during the development of the disease, and this is at least via inhibiting PP2A activity and activating GSK3β [19–21].

Medicinal plants are widely used as alternative therapeutic strategy for the prevention or treatment of many diseases in different parts of the world. *Tamarix gallica* (TG) belongs to the family of *Tamaricaceae*. The principal constituent in TG is tamarexin along with polyphenolic compounds such as flavonoids (Naringenin, Quercetin, Rhamnetin, Rhamnazin, Tamarixetin, Kaempferol, QGlcA, QGlcA-Me, KGlcA, KGlcA-Me), phenolic acids, tannins alkaloids and glycosides, and has showed some beneficial anti-Aβ aggregation and anti-diabetic effects *in-vitro* [22]. Additionally, TG was reported to possess multiple pharmacological activities such as antioxidant, anticancer, hepatoprotective, antihyperlipidemic [23,24], anti-inflammatory, antalgic [25] antimicrobial [26] and inhibitor of nephrolithiasis [27]. Interestingly TG is relatively safe as no death was observed in an acute oral toxicity study at a dose of up to 3000 mg/kg body weight of its methanolic extract in albino rats [28]. There are many studies on the antioxidative property of TG and its use in the treatment of degenerative diseases like diabetes mellites [22,23,26,29], but so far there is no report on the use of TG in the treatment of AD lesions such as tau and Aβ pathologies. In this study, we produced an HHcy AD rat model by Hcy injection. These were then supplemented with TG extract to assess the potential of TG on HHcy-induced AD-like pathological changes and the underlying mechanisms.

## RESULTS

### Phytochemical screening

As previously reported, phytochemical screening of methanolic extract of TG confirmed the presence of tannins and antioxidants including flavonoids, alkaloids and phenols. These compounds confer TG its antioxidative property and could be responsible for many of its medicinal effects.

### TG ameliorated memory deficits induced by Hcy

**Figure 1:**
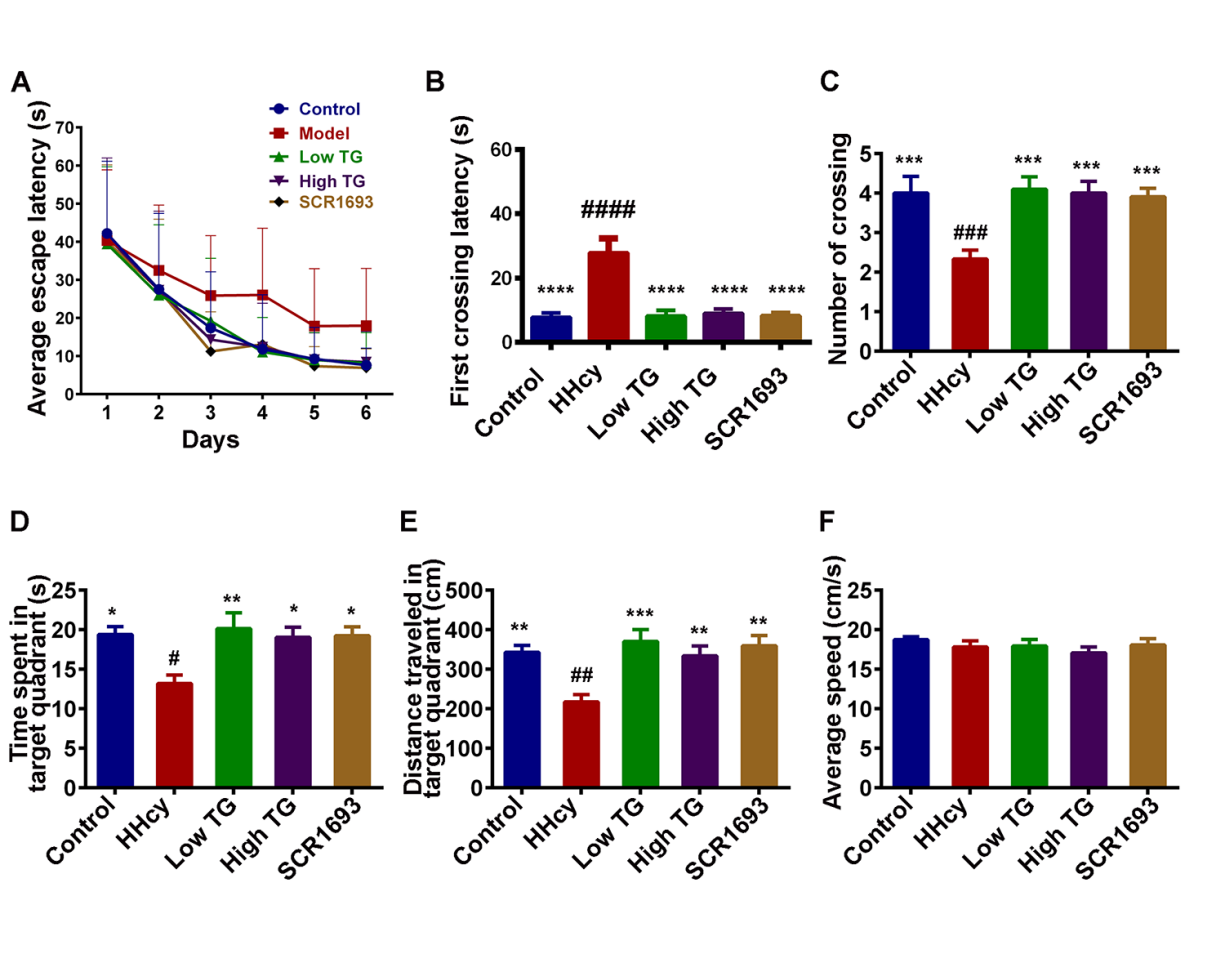
**TG treatment improved Hcy-induced learning and spatial memory impairments.** Sixty male SD rats were divided into 5 groups as Control, Homocysteine only (HHcy), Low TG treatment (Low TG), High TG treatment (High TG) and Positive Control (SCR1693) groups. The rats were subjected to 3 trials per day to find the hidden platform in the Morris Water Maze (MWM) and the memory and learning abilities of the animals were tested for 60s on the seventh day. (**A**) Escape latency to find the hidden platform in MWM for the six training days. (**B**) First crossing latency and (**C**) mean number of crossing the position of the hidden platform on the test day. (**D**) Time spent and (**E**) distance traveled in the target quadrant on the test day. (**F**) Average speed during the 60s of the test. HHcy rats showed impaired spatial learning and memory and treatment with either low or high TG rescued these impairments. The data were expressed as mean ± SEM (n = 12). # P < 0.05, ## P < 0.01, ### P < 0.001, #### P < 0.0001 versus control and * P < 0.05, ** P < 0.01, *** P < 0.001, **** P < 0.0001 versus HHcy.

Oxidative damage in the brain is a risk of cognitive impairments in humans [30]. Previous study also showed that high plasma Hcy is harmful and induced spatial memory impairments in rats [31]. Under this setting, the assessment of learning and memory functions in the spatial reference memory task revealed a marked deficit in HHcy rats compared to the controls. Like the positive control (SCR1693), the escape latency of both low and high TG doses groups was restored to normal control level (Fig.1 A). The latency to cross the position of the platform for the first time was significantly increased (Fig.1 B) while the mean number of crossing the position of the platform (Fig.1 C), the time spent (Fig.1 D) and distance covered (Fig.1 E) in the target quadrant were decreased in the HHcy rats compared with the control ones, and supplementation with TG significantly recovered these impairments. In addition, no significant difference in the swimming speed was observed among all groups, indicating that Hcy or TG does not affect motor function (Fig. 1F). These results demonstrated that TG can effectively attenuate the memory deficits induced by Hcy.

### Hcy-induced oxidative damage was restored by TG treatment

**Figure 2:**
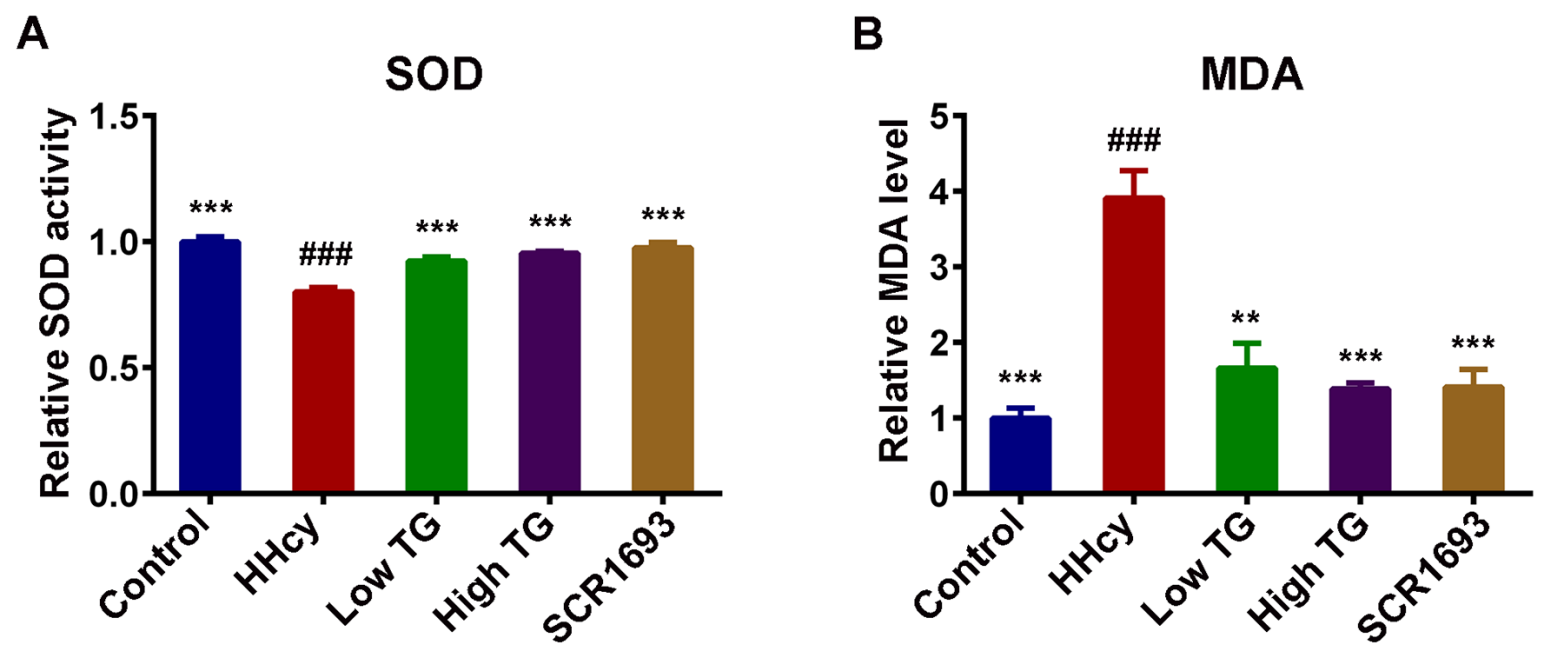
**TG attenuated Hcy-induced oxidative stress.** (**A**) Relative SOD activity and (**B**) relative level of MDA in serum. Compared with control group, HHcy rats showed decreased activity of SOD and increased level of MDA. Both treatment high and low with TG markedly increased SOD activity and decreased MDA level. The data were expressed as mean ± SEM. Data were from 3 different animals in each group (n = 3). ### P < 0.001 versus control; ** P < 0.01, *** P < 0.001 versus HHcy.

Multiple lines of evidence indicate that oxidative stress not only strongly participates in the early stage of Alzheimer’s disease prior to cytopathology but also plays an important role in inducing and activating multiple cell signaling pathways that contribute to the formation of toxic substances in the development of AD [32]. HHcy is reported to be an indicator of oxidative stress [33]. To investigate the effect of TG on oxidative stress in the present study, we employed Hcy to mimic this condition and then measured the level of malondialdehyde (MDA) and the superoxide dismutase (SOD) activity in the serum. In our previous study we reported both peripheral blood and brain oxidative stress in HHcy rat model [34]. Here we further revealed that continuous treatment with Hcy led to peripheral oxidative stress characterized by a significant increased level of MDA and a decreased activity of SOD in the serum of HHcy rats when compared with the control group (Fig.2 A, B). Meanwhile, the treatments with TG, both low and high doses, like the SCR1693, markedly decreased MDA level and increased SOD activity in serum and brought them to almost control levels. These data suggest that TG attenuated the peripheral oxidative damage induced by Hcy.

### TG rescued Hcy-induced neurodegeneration

**Figure 3:**
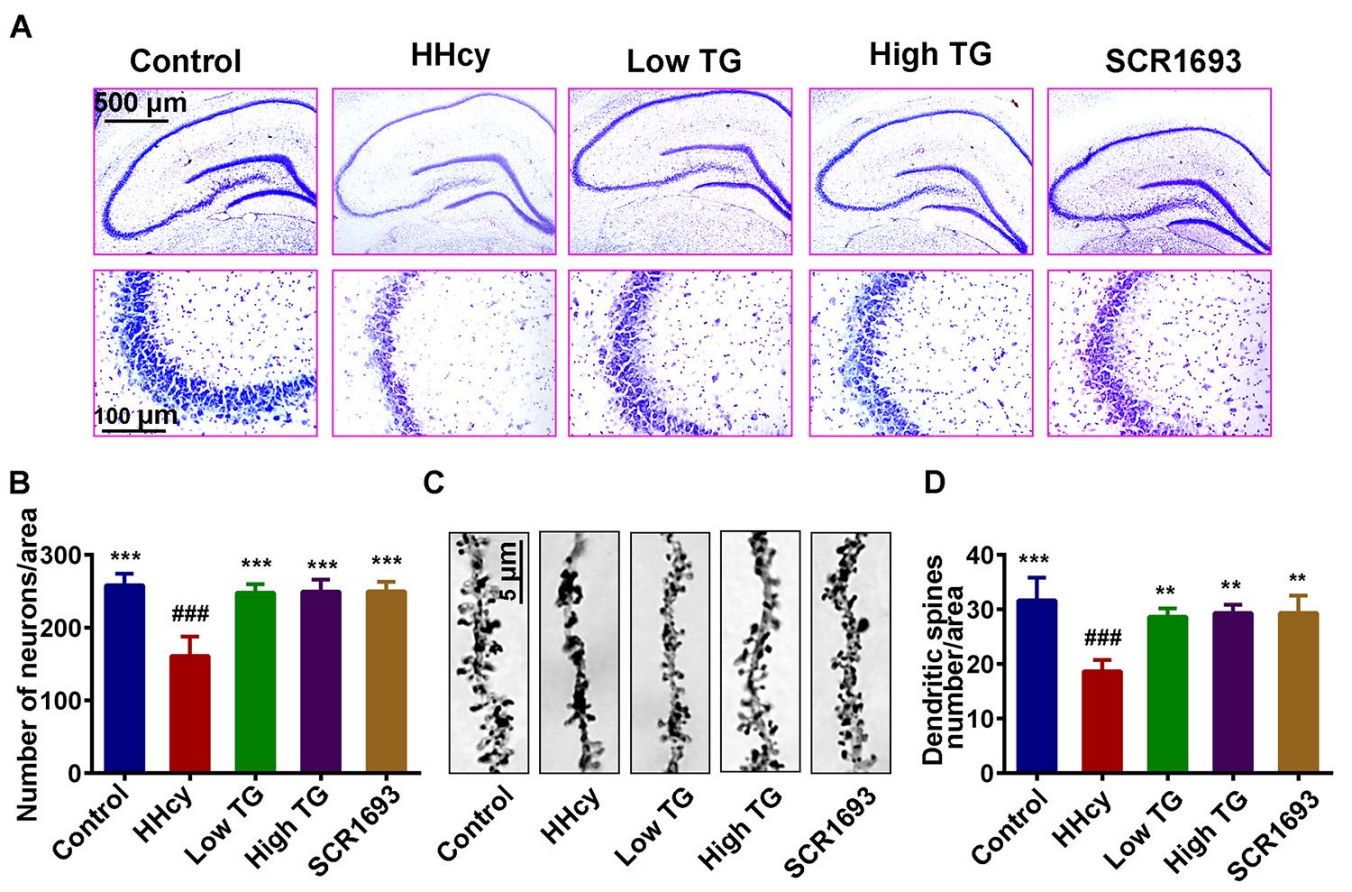
**Supplementation with TG recovered dendritic spine and neuronal loss.** After two weeks of Hcy (400 µg/kg/day) and two weeks TG treatment post-injection, the rats were sacrificed following behavioral test. (**A** and **B**) Representative Nissl staining images and the quantification of neuronal density, chart bar = 500 and 100 µm for low and high magnifications respectively. (**C** and **D**) Representative Golgi staining images and quantification of dendritic spines from randomly selected dendritic segments of randomly selected hippocampal neurons, chat bar = 5µm. HHcy animals showed neurodegeneration which was recovered following supplementation with both low and high TG doses. The data were expressed as mean ± SEM and n = 3 for both Nissl and Golgi staining. ### P < 0.001 versus control; ** P < 0.01, *** P < 0.001 versus HHcy.

Given that previous studies showed Hcy was found to induce the loss of neuronal integrity that led to neurodegeneration [35,36], and our results revealed that TG improved oxidative stress and memory impairment induced by Hcy and, we hypothesized memory impairment could be a reflection of impaired synaptic and/or neuronal plasticity. To investigate whether TG could rescue the Hcy induced neurodegeneration, we analyzed the dendritic morphology and spine density of the pyramidal neurons in the hippocampus of the rat using the Nissl and Golgi staining methods. The analysis of the Nissl staining revealed that the number of hippocampal neuronal cells was decreased following Hcy injection when compared with the control and was significantly recovered by TG treatment (Fig.3 A, B). Likewise, the average number of dendritic branches in the Golgi images was remarkably reduced following treatment with Hcy, however, the supplementation with TG rescued this deficit (Fig.3 C, D). In general, these results highlight the adverse effects of Hcy on dendritic structure and morphology of neurons and these changes were reversed by TG.

### TG preserved synaptic integrity and attenuated homocysteine induced neuro-inflammation

**Figure 4:**
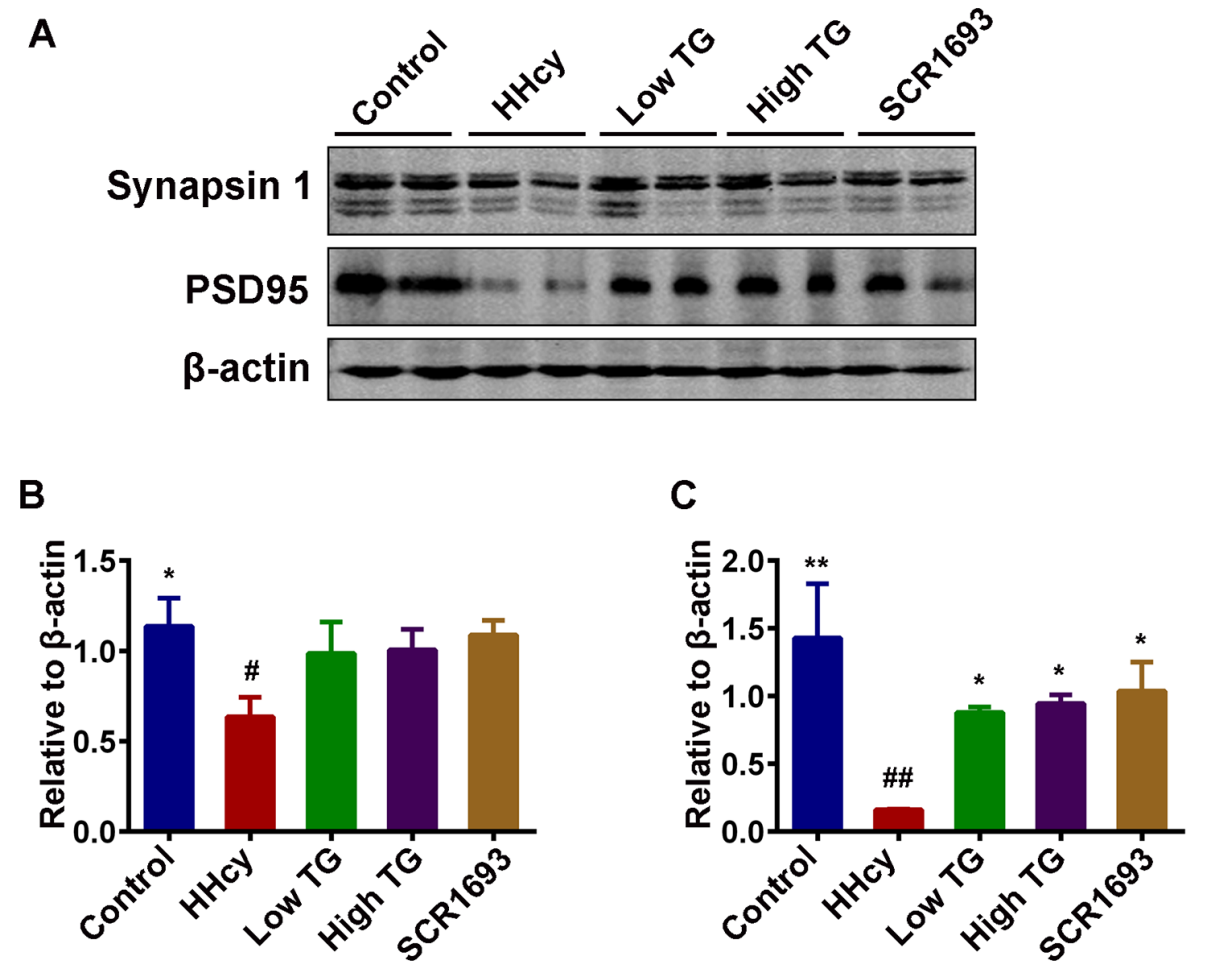
**TG supplementation attenuated the decrease of memory-related proteins.** (**A**) Levels of synapsin 1 and PSD95 were detected using western blotting technique in the hippocampus and β-actin was used as loading control. (**B** and **C**) Quantitative analysis of the blots showed that Hcy dramatically decreased the expression of these synaptic proteins in the hippocampus when compared with control group, and treatment with TG reversed these effects. The data were expressed as mean ± SEM (n = 6). # P < 0.05, ## P < 0.01 versus control; * P < 0.05, ** P < 0.01 versus HHcy.

The toxicity of Hcy to CNS neurons is widely recognized and affects both neuronal survival rate and the ability of neurons to transmit signal and to further form functional neuronal networks. Normal synaptic function relies on the stable expression of synaptic proteins, such as PSD95 in the post-synapse and synapsin 1 in the pre-synapse [37]. To further explore the potential mechanisms how TG ameliorates memory deficits induced by HHcy, we evaluated the levels of PSD95 and synapsin 1 by western blotting. We found that Hcy dramatically suppressed PSD95 and synapsin 1 levels in the hippocampus while the treatment with TG reversed these effects (Fig.4 A-C).

**Figure 5:**
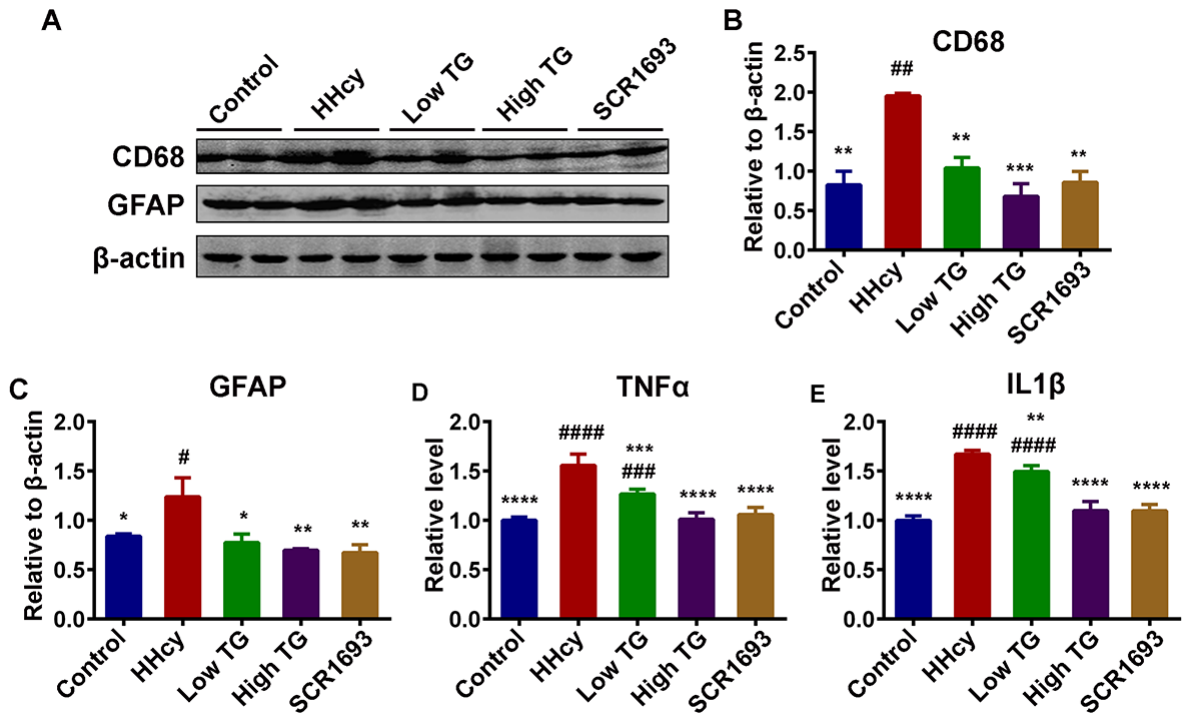
**TG downregulated neuroinflammation induced by Hcy injection in rats.** (**A-C**) Western blots and quantitative analysis of inflammatory markers CD68 and GFAP (n = 6). TG treatment decreased the Hcy-upregulated level of these markers back to control level. (**D** and **E**) ELISA results revealed an increased level of inflammatory mediators TNFα and IL1β following Hcy injection and a decrease upon TG supplementation (n = 3). The data were expressed as mean ± SEM. # P < 0.05, ## P < 0.01, ### P < 0.001, #### P < 0.0001 versus control and * P < 0.05, ** P < 0.01, *** P < 0.001, **** P < 0.0001 versus HHcy.

Homocysteine is deemed to affect microglia proliferation in *in-vitro* model. Patients suffering from severe HHcy manifest typical clinical cardiovascular symptoms as well as neurological disorders, such as cerebral atrophy, dementia and seizures [38] while STAT3 overactivation in microglial cells plays an important role in Hcy-induced microglia activation and inflammatory response, both in the brain cortex and the dentate gyrus (DG) region of the hippocampus, following ischemic injury [38]. This inflammation manifests as increased pro-inflammatory cytokine expression by resident microglia, as well as microglia proliferation, and is markedly found in the hippocampus [39]. To determine whether the improvements in cognition found in behavioral tasks following TG treatment were associated with underlying changes in hippocampal neuro-inflammation, we assess inflammatory process in the hippocampus by western blot. The protein level of CD68 which is expressed by activated microglia and GFAP by astrocytes were evaluated as accumulating evidences have shown that homocysteine can significantly increase these (GFAP, CD45) inflammatory markers [40]. Interestingly, in this setting TG attenuates neuro-inflammation by downregulating the inflammatory markers CD68 and GFAP via reducing their expression close to control level (Fig.5 A-C). To support these findings, we performed ELISA for inflammatory mediators TNFα and IL1β as it was previously reported that HHcy is associated with induction of neuro-inflammation with concomitant increase in TNFα, IL1β and IL6 levels [41]. Expectedly, we found that TG significantly downregulated both TNFα and IL1β when compared with the HHcy group (Fig.5 D, E). These together suggest that HHcy induced the observed synaptic impairments and neuro-inflammation which were effectively recovered following the treatment with TG.

### TG attenuated Hcy-induced tau hyperphosphorylation

**Figure 6:**
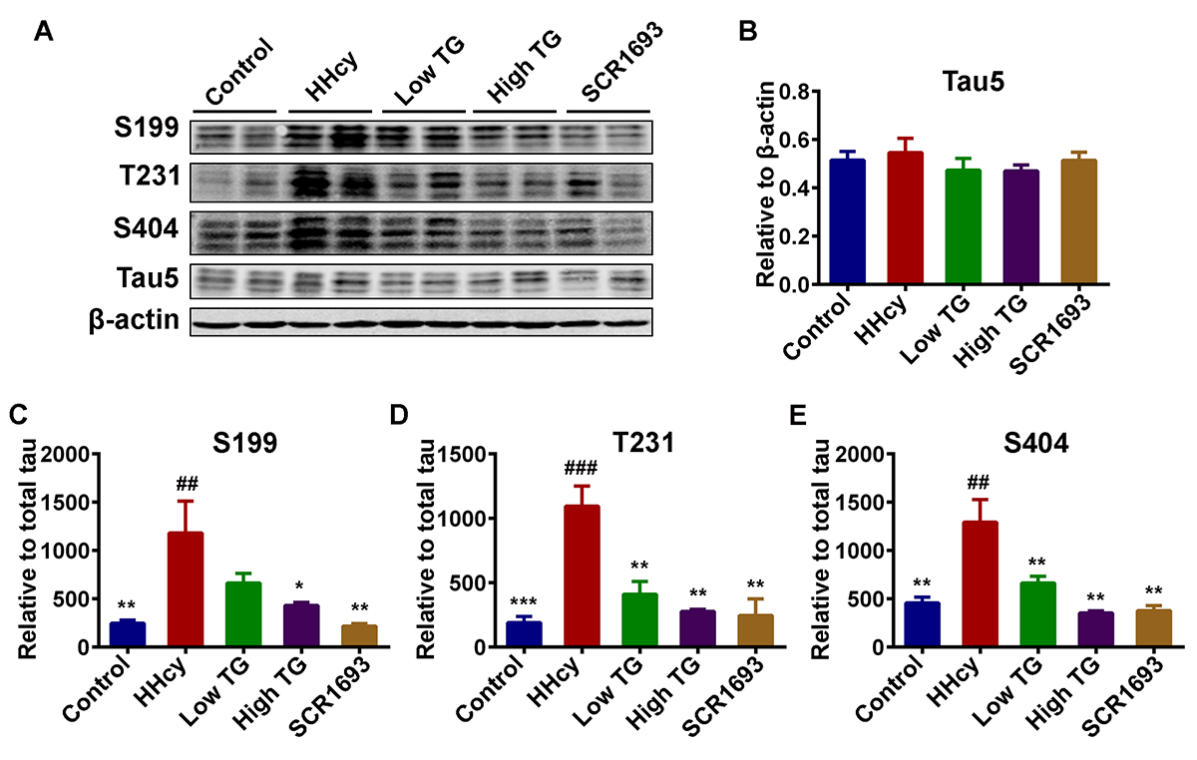
**Treatment with TG mitigated Hcy-induced tau hyperphosphorylation.** (**A**) Phosphorylation status of tau protein as measured by Western blotting in the hippocampus after Hcy treatment and TG supplementation. (**B**) Total tau (Tau5) normalized to β-actin. (**C-E**) Quantitative analysis of the blots of phosphorylated tau probed with several phosphorylated-tau antibodies normalized to total tau (Tau5). A significant decrease in tau hyperphosphorylation at several studied sites was seen following TG administration. The data were expressed as mean ± SEM (n = 6). ## P < 0.01, ### P <0.001 versus control; * P < 0.05, ** P < 0.01, *** P < 0.001 versus HHcy.

Increased and abnormal tau phosphorylation is a marker of AD and is correlated with the progression of the disease. Hyperphosphorylation and accumulation of tau protein contribute to the learning and memory deficits [42,43]. Therefore, in this study we measured the levels of phosphorylated tau by western blot. The level of total tau showed no significant difference among all groups (Fig.6 A, B). However, the levels of the phosphorylated tau at Ser199 (p-S199), Ser404 (p-S404) and Thr231 (p-T231), in the hippocampal extracts were all increased in the HHcy rats compared to the control ones, while TG treatment attenuated the Hcy-induced tau hyperphosphorylation at the above-mentioned sites (Fig.6 A-E). These data imply that TG could attenuate Hcy-induced tau hyperphosphorylation.

### TG decreased tau phosphorylation by modulating tau-related kinases and phosphatases

**Figure 7:**
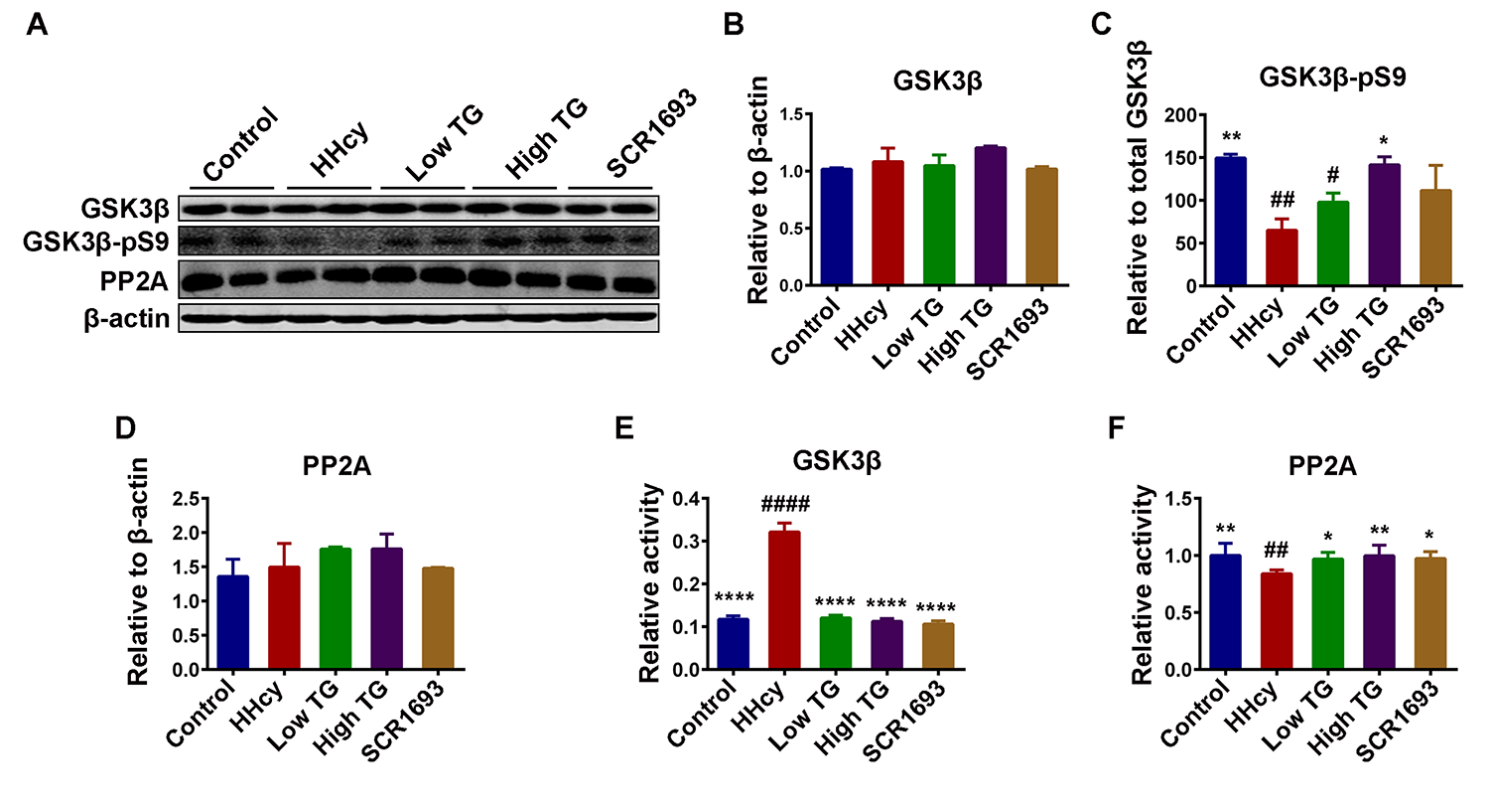
**TG decreased the phosphorylation level of tau by modulated tau related phosphatase and kinases.** (**A-D**) Western blotting and quantitative analysis of total glucose synthase kinase 3β (GSK3β), phosphorylated GSK3β at Ser9 (GSK3β-pS9) and total protein phosphatase 2A (PP2A) (n = 6). No significant difference in the total level of either GSK3β or PP2A among all groups, but the GSK3β-pS9 was significantly high in HHcy and was reduced following TG supplementation. GSK3β (**E**) and PP2A (**F**) activity tests (n = 3) revealed an increased GSK3β and a decreased PP2A activity after Hcy which were reversed by both low and high TG treatments. The data were expressed as mean ± SEM. # P < 0.05, ## P < 0.01, #### P <0.0001 versus control; * P < 0.05, ** P < 0.01, **** P < 0.0001 versus HHcy.

The actual phosphorylation status of the tau protein is regulated by kinases/phosphatases system which was found to be disrupted in AD [44]. GSK3β is one of the key kinase that phosphorylates tau [45] and is associated with oxidative stress [10]. Furthermore, studies have shown that Hcy increased AD-like tau hyperphosphorylation by inactivating PP2A via increasing inactivated phosphorylated and demethylated PP2Ac. To explore the mechanisms underlying the anti tau-phosphorylation effect of TG, the protein levels of GSK3β and PP2A, and the phosphorylation of GSK3β at Ser9 (GSK3β-pS9) were evaluated by western blot (Fig. 7A). The results showed that the levels of total GSK3β and PP2A were comparable among all groups (Fig.7 B and D), however the GSK3β-pS9, which is indicative of its inhibition, was decreased in the HHcy rats compared with the controls and was significantly recovered by the TG treatments (Fig.7C). This finding is further confirmed by the ELISA results that showed an increased activity of GSK3β (Fig.7 E) and a decreased activity of PP2A in the HHcy rats (Fig.7 F).

**Figure 8:**
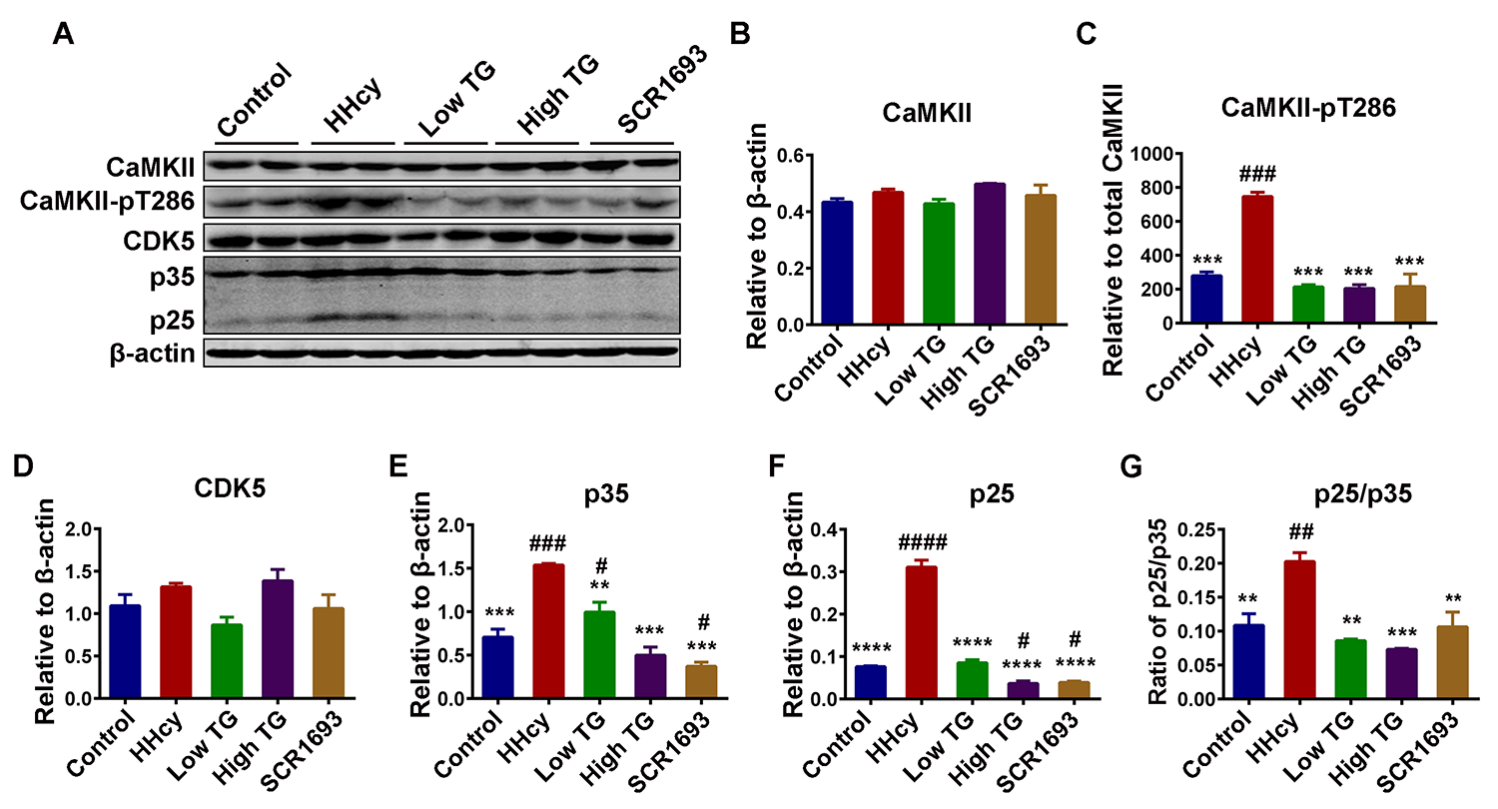
**TG decreased tau hyperphosphorylation by decreasing the activity of tau related Ca^2+^-dependent kinases CDK5 and CaMKII.** (**A**) Western blots of total Ca^2+^/calmodulin-dependent protein kinase II (CaMKII), phosphorylated CaMKII at Thr286 (CaMKII-pT286), total cyclin dependent kinase 5 (CDK5) and CDK5 activators (p35 and p25) in the hippocampal lysates. (**B-F**) Quantitative analysis of the blots, total CaMKII, CDK5, p35 and p25 were normalized to β-actin while CaMKII-pT286 was normalized to total CaMKII. (**G**) The ratio of p25/p35. Total protein levels of CaMKII and CDK5 were comparable among all groups, however their activities, as measured by increased CaMKII-pT286 for CaMKII and increase in both activators (p25 & p35) and ratio of p25/p35 for CDK5, were high in HHcy group and decrease to control level by TG treatment. The data were expressed as mean ± SEM (n = 6). # P < 0.05, ## P < 0.01, ### P < 0.001, #### P < 0.0001 versus control; ** P < 0.01, *** P < 0.001, **** P < 0.0001 versus HHcy.

By inducing oxidative stress, Hcy was also reported to activate NMDA receptors leading to calcium influx into the cells [46,47], which could activate calcium related kinases and lead to tau hyperphosphorylation. Therefore, the calcium-dependent kinases CaMKII and CDK5 were also evaluated. The total level of CaMKII and CDK5 remained comparable among all groups (Fig. 8A, B and D). On the other hand, TG supplementation significantly downregulated the phosphorylated-CaMKII (CaMKII-pT386), the active form of this kinase, which was increased in the HHcy group (Fig. 8C). Calpain is a Ca^2+^-dependent protease which is known to cleave p35, a partial activator of CDK5 into its most active form p25 also leading to tau hyperphosphorylation [40]. Since TG rescued the Hcy-induced oxidative stress we therefore, in this setting, speculated that TG may likely inhibit or at least decrease the conversion of p35 into p25. As expected, the western blot result showed an increased in both p35 and p25 in the HHcy groups compared to the control, while TG treatment restored them to the levels comparable with the control (Fig. 8A, E and F). The ratio of p25/p35 indicating the increased activity of calpain and increased activation of CDK5 as a result of intracellular Ca^2+^ influx was also higher in the HHcy animals and was also rescued by the TG supplementation (Fig. 8G). Taken together, these results strongly suggest that TG treatment decreased tau phosphorylation by decreasing the activity of tau-related kinases while increasing that of phosphatase probably via decreasing oxidative stress.

### TG reduced Aβ production induced by Hcy

**Figure 9:**
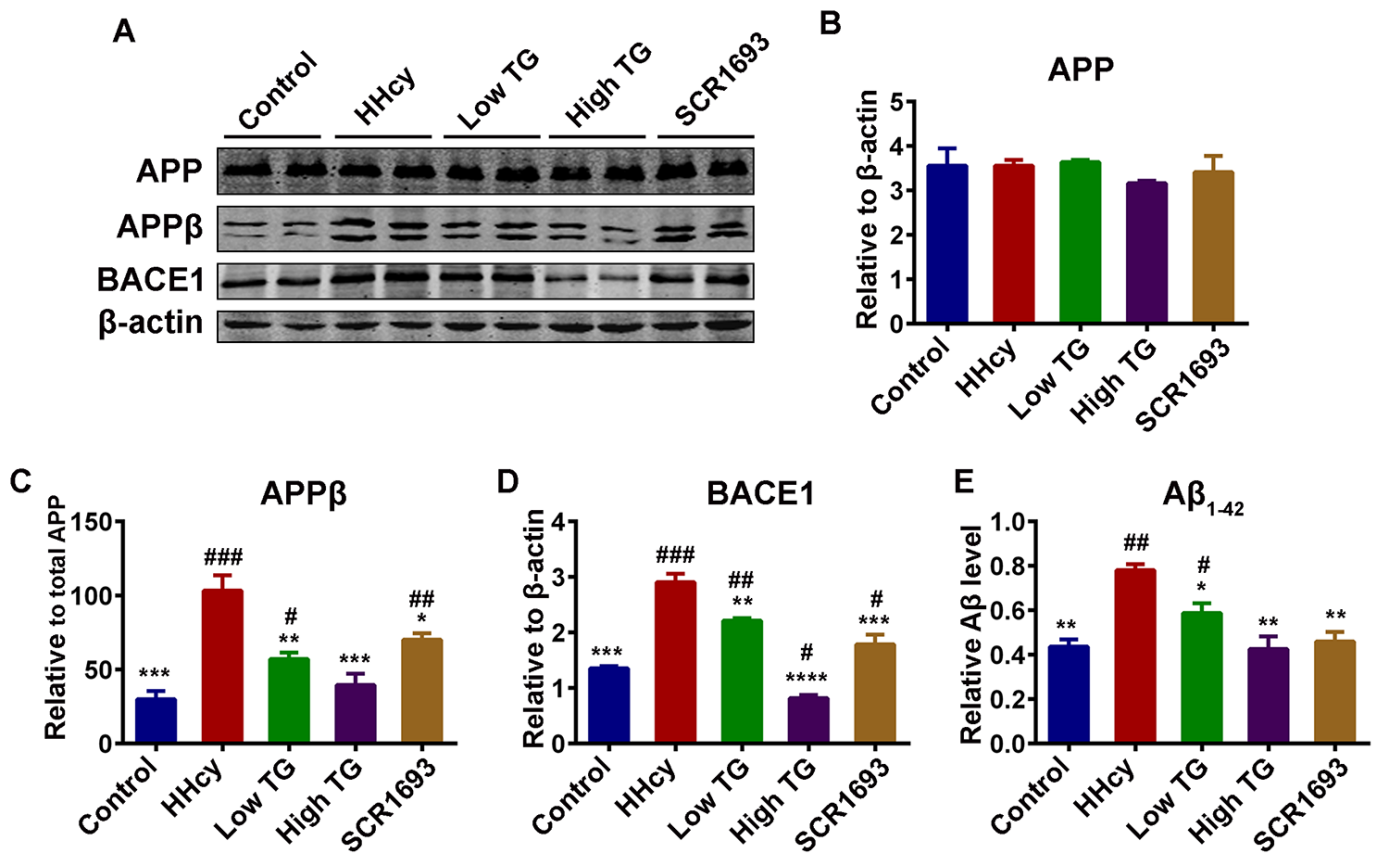
**Supplementation with TG attenuated Hcy-induced Aβ pathology.** (**A**) Levels of total amyloid precursor protein (APP), the β APP cleaving enzyme 1 (BACE1) cleaved APP [APPβ] and the total BACE1 were estimated by western blot (n = 6). (**B-D**) Quantification of the western blot results with APP and BACE1 normalized to β-actin and APPβ normalized to total APP. Total APP level remained unchanged, however the APPβ and BACE1 were high in the HHcy group while they were similar to control in the TG treatments groups. (**E**) ELISA assay for Aβ_1-42_ (n = 3) with significantly low levels of Aβ_1-42_ in the control, positive control and TG groups compared to level found in the HHcy group. The data were expressed as mean ± SEM. # P < 0.05, ## P < 0.01, ### P < 0.001 versus control; * P < 0.05, ** P < 0.01, *** P < 0.001, **** P < 0.0001 versus HHcy.

Amyloid-β peptides are a result of the action of BACE1 on APP leading to APPβ, which is then cleaved by γ-secretase to produce Aβ peptides [48]. As reported previously, injecting the rats with Hcy for 14 days induced Aβ pathology resulting in increased Aβ production [34]. We then investigated the effects of supplementation with TG on Aβ pathology induced by Hcy in the hippocampus. The western blot result revealed no significant difference in the total level of APP (Fig.9 A, B), but the APPβ protein level was significantly high following Hcy treatment when compared with the control (Fig.9 A, C). Consistent with this, the protein level of BACE1 was increased in the HHcy group (Fig.9 A, D). The treatment with TG extract significantly rescued the increase in both APPβ and BACE1 (Fig.9 A-D). To confirm this result, ELISA was carried out for Aβ_1-42_ and the findings showed a similar pattern with the western blot result (Fig.9 E). These data collectively indicate that TG effectively attenuated the Aβ induced pathology in the treated animals.

## DISCUSSION

Alzheimer’s disease is characterized pathologically by numerous Aβ plaques and NFTs in selective brain regions following a specific distribution pattern. AD is a neurodegenerative disease and the most common form of dementia in old people. Oxidative stress plays a critical role in the development of the disease [19,20,49] and Hcy is an independent and sufficient established environmental AD risk factor [13,15,50,51] by inducing oxidative stress. Epidemiological studies revealed that more than 40% of AD patients were found to have high plasma Hcy levels and these patients displayed more rapid neural atrophy than those with normal Hcy levels [52]. In line with previous studies [29] we also found that TG decreases oxidative stress. The behavioral test revealed that TG improved the Hcy-induced memory impairment. Moreover, TG antagonized tau hyperphosphorylation, decreased Aβ production, attenuated synaptic impairments and neuroinflammation induced by HHcy. In a similar setting with our study, SCR1693, a synthesized hybrid compound composed of an acetylcholinesterase inhibitor (AChEI) and a calcium channel blocker (CCB) was previously found to improve memory impairments [53,54], cell death and hippocampal neuron loss [55], inhibited Aβ production and induced tau dephosphorylation [35]. Therefore, we used this compound as a positive control.

Many studies have been conducted on TG and revealed that this plant is a good source of alkaloids, flavonoids and other polyphenols [22,25,29] which are responsible for its antioxidative properties [22,26,28]. Here also the phytochemical screening confirmed the presence of alkaloids, flavonoids, phenols and tannins. As in our previous and other studies, we found that Hcy injection increased MDA level and decreased SOD activity, indicating an increase in oxidative stress. TG extract significantly rescued these effects in the serum of treated animals. These observed effects could be attributed to the high content of antioxidants from TG such as flavonoids like Naringenin, Quercetin, Rhamnetin, Rhamnazin Tamarixetin, Kaempferol, QGlcA, QGlcA-Me, KGlcA, and KGlcA-Me [6,22]. Increase in oxidative stress markers was correlated with a decline in cognitive ability [56]. In the present study, when compared with control group, the HHcy rats showed impaired memory and learning abilities which could be associated with increased oxidative stress seen in this group of animals. These alterations were significantly recovered by the TG supplementation, suggesting that TG might attenuate memory and learning deficits caused by Hcy.

AD is a neurodegenerative disease characterized by loss of neurons. Memory is the reflection of good and functional neuronal and synaptic plasticity and integrity. In this study we observed that following the treatment with Hcy, there was a significant reduction in the number of neuronal cells as well as the number of dendritic spines, indicating neurodegeneration. These alterations were rescued by supplementation with TG extract. Normal synaptic function requires the stable expression of presynaptic and postsynaptic proteins [37] and this is essential for learning and memory [57]. The results from the present study indicated that Hcy dramatically suppressed the protein levels of both PSD95 and synapsin 1 in the hippocampus and treatment with TG reversed these effects to levels comparable with the control. These results indicate that TG ameliorated learning and memory probably by preserving neuronal and synaptic integrity.

Amyloid-β aggregates and intracellular NFTs, the main markers of AD, have both been closely associated with activated microglia. Furthermore, Hcy was shown to significantly increase pro-inflammatory markers like GFAP and CD45, suggesting an activation of astrocytes and microglia respectively [40]. A similar phenomenon characterized by increased GFAP and CD68 as shown by western blot was observed in our study. Withal, these changes were restored by the TG treatment. High Hcy level in rodent models was shown to induce cognitive decline, systemic vascular inflammation and atherosclerosis [58]. Evidences have shown that deficits in cognitive and synaptic functions, including attenuation of LTP, were related to increased concentration of inflammatory cytokines such as IL1β [49]. Previous studies also showed that HHcy may result in neuroinflammation as indicated by elevated levels of IL1β, TNFα and IL6 in mice brain tissue, and these inflammatory mediators were all shown to independently result in cognitive impairments [41,59,60]. To further confirm the anti-inflammatory potential of TG, we measured the levels of IL1β and TNFα by ELISA. Interestingly, the findings from ELISA were in line with the western blot as the supplementation with TG significantly downregulated these inflammatory markers (IL1β and TNFα) that were elevated by the Hcy injection. It is important to note that one potential link between inflammatory process and AD is the fact that inhibition of IL1 signaling reduces the activity of several tau kinases in the brain, including CDK5/p25, GSK3β and p38-MAPK, and reduces phosphorylated tau level [60]. Conclusively, these results indicate that TG might improve learning and memory via decreasing inflammatory mediators, the downstream AD effects.

Oxidative stress triggers the inactivation of PP2A [56], and calcium influx due to activation of NMDA receptors, as a result of oxidative stress, may lead to calpain activation which further induces the cleavage of the inhibitory domain (Ser9) of GSK3β. Calpain can also cleave the activator of CDK5 p35 into its most active form p25 [40]. Together, all of these factors increase in the activities of the main tau kinases (GSK3β and CDK5) resulting in tau hyperphosphorylation. Here we observed an increase in the protein levels of phosphorylated tau at several sites including p-S199, p-S404 and p-T321, which was restored to the control levels following TG treatment. These findings are in line with the results from the Hcy treatment, which decreased GSK3β-pS9 and increased p35 and p25 (CDK5 activators), and the use of the TG supplementation that reversed the changes induced by the Hcy treatment. Moreover, we observed an upregulation of the p25/p35 ratio, an indication of calpain activation leading to a more active status of the CDK5. Interestingly, p25/CDK5 inhibition has been reported to attenuate tauopathy in a model of frontotemporal dementia [61]. This could be a possible pathway via which TG attenuated tau hyperphosphorylation. Also, p25/CDK5 mediates Aβ-induced hyperphosphorylation of tau and this further increases p25 generation and CDK5 hyperactivation forming a feed forward loop. Hyperactivated CDK5 (p25/CDK5) could also facilitate or prime tau phosphorylation by other kinases, such as GSK3β [62]. Moreover, the GSK3β and PP2A activity tests revealed an increase in GSK3β and a decrease in PP2A activities in the animals injected with Hcy when compared with the control and TG and SCR1693 treatment groups. CaMKII is another calcium-dependent kinase and can be auto-phosphorylated in the presence of calcium [63]. As increased p25/p35 ratio oberserved in our study suggested calpain activation and therefore increased intracellular Ca^2+^, we measured the total and phosphorylated CaMKII. Interestingly, the total CaMKII was not changed but the CaMKII-pT286 was significantly higher in the HHcy rats than that in the controls, indicating an increased activity of this kinase which might at least contribute to the tau phosphorylation. These changes were overcome following TG treatment. Together these results stipulate that TG rescued the Hcy-induced tau hyperphosphorylation via decreasing the activities of tau related kinases and increasing PP2A activity.

Amyloid-β aggregation is one of the markers of AD in addition to p-tau, and Aβ pathology might possibly occur even before p-tau. It has been reported that elevated levels of Aβ_1 - 40_ and Aβ_1 - 42_ were associated with increased levels of oxidation products from proteins, lipids and nucleic acids in AD hippocampus and cortex [64]. Oxidative stress increased the expression of presenilin 1 and BACE1 and its activity [65]. Here in, the HHcy rats had increased APPβ as the result of the increased BACE1 as well as increased Aβ production which was confirmed by the Aβ ELISA. These alterations were eliminated by the TG supplementation, especially in the high dose treatment group. Moreover, GSK3β is known to promote the amyloidogenic processing of APP by forming a complex with presenilins and promoting their γ-secretase activity [66,67]. Our data showed that TG may attenuate Aβ production through the inhibition of GSK3β activity and the reduction of the γ-secretase activities. Also, it is known that BACE1 is the rate-limiting enzyme in processing APP to Aβ and we reported increased protein levels of BACE1, APPβ and Aβ in the Hcy treated animal, which were significantly reduced following treatment with TG. TG treatment in the present study may likely be attributed to its antioxidants content, especially flavonoids which are among the most abundant phytochemicals constituent of TG.

It has been reported that synaptic loss can be recovered by decreasing BACE1 in a p25/CDK5 model of neurodegeneration [68]. We also reported an increase in BACE1 protein level which was rescued by TG, stipulating that the preservation effect of TG on synaptic integrity might in part be due to its ability to decrease BACE1 level. The anti-AD activity of flavonoids has been believed to originate from their antioxidative activity and/or β-sheet recognition due to their hydrophobicity and planarity [69,70]. Furthermore, reports have shown that TG also contains catechol-type flavonoids which are known to inhibit the aggregation of Aβ_1-42_, in a reaction where a catechol structure is autoxidized to form an *o*-quinone on the B-ring, followed by the formation of the *o*-quinone-Aβ_1-42_ adduct targeting Lys residues at positions 16 and 28 of Aβ_1-42_ [69]. Moreover, catechol flavonoids are more potent suppressors of Aβ aggregation with lower IC50 than the non-catechol type and QGlcA was shown to be the most potent anti-amyloidogenic flavonoid in TG [22]. Quercetin, one of the strongest antioxidant flavonoids in TG, is another important bioactive compound and acts mainly by scavenging reactive oxygen species (ROS) [71]. In addition, it exerts several other pharmacological effects, such as anti-inflammatory, anti-amyloidogenic, anti-cancer and antiviral [72,73]. The observed effect of TG on Aβ metabolism in our present study was correlated with a previous *in vitro* study showing the inhibitory activities of antioxidant flavonoids from TG on Aβ aggregation in degenerative diseases like AD and type 2 diabetes mellitus [22]. These observed effects are also consistent with previously reported anti-Aβ aggregation of flavonoids, caffeoylquinic acids and acteoside [69,74,75]. Taken together, our findings highlighted the anti-Aβ pathology property of TG via decreasing BACE1 protein level and possibly via decreasing its aggregation.

In summary, the results from the present study are consistent with the fact that Hcy is a strong independent risk factor of AD, as Hcy induces memory and behavioral deficits, oxidative stress, neuroinflammation, synaptic and neuronal impairments, tau hyperphosphorylation and Aβ pathology, which were rescued by the TG supplementation, especially at high dose. Most of these effects of TG could be attributed to its antioxidative property particularly due to the presence of different types of flavonoids. The previously reported anti-aggregation property of TG and the catechol-type flavonoids in TG might play a role in decreasing Aβ production via the *o*-quinone-Aβ adduct formation. TG is a promising candidate compound for treating AD related pathologies due to the naturally occurring phytochemicals it contains.

## MATERIALS AND METHODS

### Reagents

The primary antibodies employed in this study and their properties are listed in Table 1. Secondary antibodies for Western blotting were from Amersham Pharmacia Biotech (Little Chalfort, Buckinghamshire, UK). DL-Hcy was from Sigma Chemical CO (St. Louis, MO, USA) and was dissolved in saline to the final concentration of 400 μg/ml immediately before injection.

**Table 1:** Antibodies employed in this study.

**Antibody**	**Specific**	**Cat Number**	**Type**	**Dilution**	**Source**
pT231	Phosphorylated tau at Thr231	11110	pAb	1:1000 for WB	Signalway Antibody CollegePark, MD, USA
pS199	Phosphorylated tau at Ser199	44734G	pAb	1:1000 for WB	Thermo Fisher
pS404	Phosphorylated tau at Ser404	11112	pAb	1:1000 for WB	Signalway Antibody CollegePark, MD, USA
Tau-5	Total tau	ab80579	mAb	1:1000 for WB	Abcam, Cambridge, MA, USA
PP2AC	PP2A catalytic subunit	2038	pAb	1:1000 for WB	Cell Signaling Danvers, MA, USA
Synapsin-1	Synapsin-1 C-term	AB1543	pAb	1:1000 for WB	Millipore, Temecula, CA, USA
PSD95	PSD95 N-terminal	507	pAb	1:1000 for WB	Cell Signaling Danvers, MA, USA
Gsk3β serine 9	Phosphorylated GSK3 at Ser9	9323	pAb	1:1000 for WB	Cell Signaling Danvers, MA, USA
GSK-3β	Total GSK-3β	12456	mAb	1:1000 for WB	Cell Signaling Danvers, MA, USA
CaMKII	Total CaMKII	3362	pAb	1:1000 for WB	Cell Signaling Danvers, MA, USA
p-CaMKII	Phosphorylated CaMKII at Thr286	3361	pAb	1:1000 for WB	Cell Signaling Danvers, MA, USA
β-actin	Total actin	ab6276	mAb	1:1000 for WB	Abcam, Cambridge, MA, USA
P35/P25	Total p35/p25	sc-820	pAb	1:1000 for WB	Santa Cruz, CA, USA
CDK5	Total CDK5	sc-6247	mAb	1:1000 for WB	Santa Cruz, CA, USA
CD68	Anti-human CD-68 Clone KP-1	Ab955	mAb	1:1000 for WB	Abcam Cambridge, MA, USA
GFAP	Total GFAP	3670	mAb	1:500 for WB	CST
BACE1	Total BACE1	5606S	pAb	1:1000 for WB	Cell Signaling Danvers, MA, USA
APP	Full length of total APP	2452S	pAb	1:1000 for WB	Cell Signaling Danvers, MA, USA
APPβ	Anti-human sAPPβ	18957	pAb	1:1000 for WB	IBL

Mono-, monoclonal; poly-, polyclonal; WB, Western blotting; p, phosphorylated

### Plant preparation

The aerial part of TG was ordered from Yisan (Baoding, China). The plant material was grinded to powder, and 2.5 g of the dried powder was macerated in 25 mL of 80% methanol for 24 hours with continuous shaking. Extracts were filtered through a filter paper and evaporated under reduced pressure at 35^o^C using rotary vacuum evaporator. The filtrate was kept at -80^o^C until use.

### Phytochemical Screening

The phytochemical screening was carried out as described by Tasneef Ahmad et.al [76]. Briefly to test for presence of flavonoids compound, extract of TG was treated with 10% NaOH solution. Formation of intense yellow color indicates the presence of flavonoids. To test for phenolic compounds, the extract (500 mg) was dissolved in 5 ml of distilled water. To this, few drops of neutral 5% ferric chloride solution were added. A dark green color indicates the presence of phenolic compounds. To test for the presence of tannins, to 0.5 ml of extract solution 1ml of water and 1- 2 drops of ferric chloride solution were added. A blue color was observed for gallic tannins and green black for catecholic tannins. For the presence of Alkaloids about 0.2 g of the extracts was warned with 2% H_2_SO_4_ for two minutes, filtered, and few drops of Dragendroff’s reagent were added. Orange red precipitate indicates the presence of alkaloids.

### Animals grouping and treatment

Sixty male Sprague-Dawley rats (8 weeks old, 250±25 g), supplied by the Experimental Animal Central of Tongji Medical College, Huazhong University of Science and Technology, were housed 6 animals per cage with free access to food and water under a 12:12 hours reversed light-dark cycle. The rats where divided into 5 groups of 12 animals each. For 14 days, 48 rats were injected with intravenous (IV) Hcy 400 μg/kg/day through vena caudalis, while the remaining 12 rats were injected with 0.25 ml IV normal saline and served as control. Following the Hcy injection the animals were given intragastric (IG) treatment for another 14 days. Among the 48 Hcy injected rats 12 were used as HHcy AD rats model and were given IG normal saline. Two groups of 12 rats each were treated with IG gavage of TG 100 and 300 mg/kg/day as low and high doses respectively. The remaining 12 rats were given IG SCR1693 as positive control. The control group was given 1 ml IG normal saline. The injection was performed each day from 9:00 am to 12:00 am and the animals were killed 24 hours after the final treatment following behavioral test.

### Western Blotting

Hippocampi removed from the brain were homogenized in a buffer containing Tris-Cl (pH 7.6) 10 mmol/L, Na3VO4 1 mmol/L, NaF 50 mmol/L, benzamidine 1 mmol/L, edetic acid 1 mmol/L, and PMSF 1 mmol/L. Three volumes of the homogenized tissue were added to one volume of a buffer containing Tris-Cl (pH 7.6) 200 mmol/L, 8% sodium dodecyl sulfate (SDS), and 40% glycerol, and extracts were boiled in a water bath for 10 min. The lysates were sonicated briefly and centrifuged at 12,000 x g for 5min. Protein concentration of the supernatants was measured by the bicinchoninic acid (BCA) Protein Assay Kit (Pierce, Rockford, IL, USA). The proteins were separated by SDS-polyacrylamide gel electrophoresis (10% gel) and transferred to a nitrocellulose membrane. After blocking in 3% nonfat milk for 1hour at 25°C, the membranes were incubated with primary antibodies at 4°C overnight. The blots were then incubated with anti-mouse or anti-rabbit IgG conjugated to IRDye™ (800CW) for 1hour at 25°C and visualized with the Odyssey Infrared Imaging System (LI-COR bioscience’s, Lincoln, NE, USA).

### Morris water maze test

The maze is made up of a circular black painted pool, filled with water colored with nontoxic black ink. A platform, 12 cm in diameter, was submerged 1.5 cm below the surface of the water in one of the 4 imaginary quadrants. For a period of 6 consecutive days, the rats were trained to find the hidden platform in the water maze with 3 trials per day with a 30 min interval from 8 am to 2 pm. The rat is placed in the middle of one of the three other quadrants facing the wall of the pool when it starts each trial, and the trial ended when the animal climbed on the platform. Each time the rats were allowed for a maximum of 60 seconds to search for the platform, after which if they did not find the platform, they were gently guided to the platform and were left to stay on the platform for 30s. The swimming pathway and escape latency of the rats to find the hidden platform were recorded by Noldus video tracking system (Ethovision, Noldus Information Technology, Holland). The learning and memory were tested on the seventh day. The rats were allowed to search the position of the removed platform for 60 seconds. The longer a rat stayed in the target quadrant, the better it scored in the spatial memory.

### Measurement of serum Superoxide Dismutase (SOD)

This test is based on the ability of the xanthine-xanthine oxidase system to inhibit the oxidation of hydroxylamine. Nitrite is produced following oxidation of hydroxylamine and has an absorbance at 550 nm. The amount of SOD that reduces the absorbance at 550 nm by 50% is equivalent to one unit of SOD activity. After anesthesia, blood samples were collected from the orbital arteries then immediately centrifuged at 5000 x g for 10 min at 4°C. The supernatants (serum) were used for SOD test. Total superoxide dismutase activity in the serum was estimated with a kit from Jiancheng Bioengineering Institute (Central Road, Nanjing, China), based on the ability of the xanthine-xanthine oxidase system to inhibit the oxidation of hydroxylamine.

### Measurement of serum Malondialdehyde (MDA)

Lipids peroxidation produce malondialdehyde (MDA) which level is measured by colorimetric method. After anesthesia blood samples were collected from the orbital arteries then immediately centrifuged at 5000 x g for 10 min at 4°C and the supernatants were used for MDA test. MDA, a metabolite of lipid peroxides, was used as an indicator of lipids peroxidation. MDA level in the sample was estimated using a kit from Jiancheng Bioengineering Institute (Central Road, Nanjing, China) by measuring the MDA formed by the thiobarbituric acid (TBA) reaction. N-butanol was used for extraction during this process. An equal amount of N-butanol was added into each tube of a mixed solution prepared according to the instructions, and then centrifuged the newly mixed solution at 10,000 x g for 10 min at 4°C, and the supernatants were measured at 532 nm.

### ELISA for Aβ_1-42_, IL1β and TNFα

A sandwich ELISA kit was used to measure the Aβ_1-42_, IL1β and TNFα level according to manufacturer’s protocol (Elabscience). Briefly hippocampal tissue pieces from rats were homogenized in PBS at a ratio of tissue weight (g): PBS (mL) volume of 1:9 with a glass homogenizer on ice. To further break down the cells, the lysate was sonicated with an ultrasonic cell disrupter. The homogenates were then centrifuged for 5 min at 5000 x g to get the supernatant. The protein concentration was assessed by BCA (Pierce, Rockford, IL, USA) method, and 100 µl containing 250 µg of proteins from the soluble fractions of either Aβ_1-42_, IL1β or TNFα were incubated in the microplates pre-coated with their corresponding antibodies for 90 min at 37^o^C. Wells were washed and incubated with a biotinylated detection antibody for 60 min at 37^o^C. Samples were washed then incubated with an HRP-labeled conjugate for 30 min at 37^o^C, washed again and incubated with a substrate reagent for 15 min at 37^o^C and finally the stop solution was added. The optical density was measured at a wavelength of 450 nm.

### GSK3β activity assay

GSK3β activity in hippocampal lysate was measured according to the manufacturer’s protocol (GENMED, USA). Briefly all reagents were brought to room temperature. Assay buffer (65 µl) were pipetted into separate cuvettes, then10 µl of enzyme mixture, 10 µl of reaction buffer and 10 µl of substrate were added to each well. Next the cuvettes were allowed to equilibrate to assay temperature (30°C) for 3 min. The reaction was started by the addition of 5 µl sample containing 50μg of protein while dilution buffer was added to the blank cuvettes. The plates were continuously red at 340 nm using a plate reader. The data were recorded at 1 min time intervals for 5 min.

### PP2A activity assay

PP2A activity in the brain hippocampi homogenate was measured using the phosphatase kit according to the manufacturer’s procedure (Promega). Briefly phosphate standard was diluted with phosphate free water at the ratio of 1:20 to generate a solution containing 50pmol/µl, then wells, containing 0, 100, 200, 500, 1000, 2000 pmol free phosphate and 1 x reaction buffer, were prepared in 50µl and used for standard curve. Appropriate reaction premix solutions excluding enzyme samples were directly prepared in a 96 well plate as follow:10µl of PPase 5 x reaction buffer, 5µl of 1Mm phosphopeptide and allowed at 4^o^C for 3 min. The reaction was started by adding 35µl of the enzyme sample to the well then incubated for 40 min at 37^o^C. The reaction was terminated by adding 50µl of Molybdate Dye/Additive mixture and left at room temperature for 15min and red at 630 nm.

### Nissl staining

The rats were deeply anesthetized by chloralhydrate (1 g/kg) injection and perfused through the aorta with 400 ml of 0.9% filtered saline followed by 400 ml phosphate buffer containing 4% paraformaldehyde. Their brains were dissected out and post-fixed in the same 4% paraformaldehyde solution overnight at 4^o^C. The fixed brains were sliced coronally through the area of hippocampus and cortex to 30 µm sections using vibratome (VT 1000 s, Leica, Germany). After 30 µm coronal sections were cut, gelatin-coated slides were used to mount the sections and the mounted sections were incubated in Cresyl violet for 10 min at 25^o^C, dehydrated through 50%, 75%, 95% and 100% alcohol, then cover-slipped with neutral balsam following clearance in xylene and pictures were taken using a light microscope (Olympus BX60, Tokyo, Japan).

### Golgi staining

After sacrifice and perfusion with 9% saline, the brains were removed and placed in the Golgi solution (1 g potassium chromate, 1g mercuric chloride, 0.8 g potassium chloride and 100 ml double-distilled water) for three weeks while changing the solution every two days. Sections of 80 μm thickness were cut and placed on gelatin-coated glass slides. Slides were rinsed with double-distilled water after which they were incubated in ammonium hydroxide for 50 min. The slides were washed with distilled water, then incubated for 30 min in a black and white film developer, diluted 1:9 with water, in the dark. Following a rinse in distilled water, the slides were dehydrated in subsequent concentrations of alcohol 50, 70 and 95% for 1 min each, then in 3 changes of 100% alcohol for 5 min each. After being cleared in CXA (chloroform, xylene, alcohol at 1:1:1) solution for 15 min, slides were mounted with neutral balsam and cover-slipped and allowed to dry for 24 hours then visualized under a light microscope. The images were taken using a light microscope (Olympus BX60, Tokyo, Japan).

### Statistical Analysis

Data were expressed as mean ± SEM and analyzed using GraphPad Prism 6.05 for Windows-statistical software (GraphPad Software, La Jolla California USA, www.graphpad.com). Statistical difference was determined by one-way ANOVA procedure followed by Student-Newman-Keuls post hoc test with 95% confidence and Student’s t-test. A level of p < 0.05 was accepted as statistically significant.
